# Neuromyelitis Optica Spectrum Disorder Treatment—Current and Future Prospects

**DOI:** 10.3390/ijms22062801

**Published:** 2021-03-10

**Authors:** Marta Waliszewska-Prosół, Justyna Chojdak-Łukasiewicz, Sławomir Budrewicz, Anna Pokryszko-Dragan

**Affiliations:** Department of Neurology, Wroclaw Medical University, 50-556 Wroclaw, Poland; justyna.ch.lukasiewicz@gmail.com (J.C.-Ł.); slawomir.budrewicz@umed.wroc.pl (S.B.); annapd@interia.pl (A.P.-D.)

**Keywords:** neuromyelitis optica spectrum disorder, demyelinating diseases, aquaporin 4, autoimmune humoral response, treatment

## Abstract

Neuromyelitis optica (NMO) is an immune-mediated demyelinative disorder of the central nervous system affecting mainly the optical nerves and the spinal cord. The recurrent course of the disease, with exacerbations and incomplete remissions, causes accumulating disability, which has a profound impact upon patients’ quality of life. The discovery of antibodies against aquaporin 4 (AQP4) and their leading role in NMO etiology and the formulation of diagnostic criteria have improved appropriate recognition of the disease. In recent years, there has been rapid progress in understanding the background of NMO, leading to an increasing range of treatment options. On the basis of a review of the relevant literature, the authors present currently available therapeutic strategies for NMO as well as ongoing research in this field, with reference to key points of immune-mediated processes involved in the background of the disease.

## 1. Introduction

Neuromyelitis optica (NMO), also known as Devic’s disease, is an immune-mediated demyelinative disorder of the central nervous system (CNS). Originally, it was considered a variant of multiple sclerosis (MS) [1]. Only in 2004, with the discovery of specific IgG antibodies directed against aquaporin 4 (AQP4), considered as patognomic for NMO, did it become possible to classify this disorder as a separate entity [2].

The classical NMO phenotype involves uni—or bilateral optic neuritis and transverse myelitis. Due to the presence of additional clinical manifestations (e.g., area postrema or acute brainstem syndromes) in otherwise typical cases, a wider category of NMO spectrum disease (NMOSD) has been defined [3,4].

In 2015, Wingerchuk et al. published the international consensus on diagnostic criteria for NMOSD (Table 1). According to these, NMOSD includes: classical NMO (optic neuritis—ON + longitudinal extensive transverse myelitis—LETM), isolated ON or LETM, ON and/or LETM associated with autoimmune systemic diseases, ON and LETM accompanied by symptoms of brainstem, diencephalon or cerebral involvement, and the Asian oculospinal form of multiple sclerosis [5,6].

The seropositive form of NMOSD, defined by the presence of antibodies against AQP4, accounts for approx. 80% of cases [7]. In a proportion of seronegative cases, other pathogenic antibodies are found—against myelin oligodendrocyte glycoprotein (MOG). This component of the myelin sheath is responsible for the stability of the myelin structure and its interactions with the immune system, including the complement activation pathway.

Clinical manifestations of MOG-antibody-associated disease (MOGAD) may include the typical NMO phenotype, be limited to isolated optic neuritis (in adults) or develop into acute disseminated encephalomyelitis (ADEM—mostly seen in children). On the basis of immunological and pathological findings, it has been suggested that MOGAD be positioned as a separate entity, with its possible partial overlap with NMOD and MS [4,7].

In double seronegative NMOSD patients, undetectable low levels of antiAQP4 or antiMOG Ig are considered, but there is also the possible presence of other, yet unidentified autoreactive antibodies [6,7,8,9].

NMO prevalence is 0.5–10/100.000 in Caucasians and is the highest in the population of the Far East [10]. The disease occurs significantly more commonly in women (9:1) [6]. The mean age at onset is the fourth decade. The risk of first relapse is increased in the third trimester of pregnancy and in the postpartum period; in approximately 20–30% of patients, onset is preceded by infection or vaccination. About 3% of patients have a positive family history for NMO. Apart from NMO classified as a clinical manifestation of systemic autoimmune diseases (e.g., lupus erythematosus), it may coexist with other autoimmune conditions, including autoimmune thyroiditis, myasthenia gravis, Sjögren’s syndrome, celiac disease or primary sclerosing inflammation of the biliary tract [3,6,11].

During a relapse, the symptoms subacutely increase to reach a plateau phase and then gradually subside, usually with incomplete remission. In the majority of cases (80–90%) the course of the disease is recurrent, while isolated and seronegative forms tend to be monophasic [6]. Accumulation of residual symptoms after subsequent relapses results in progressive disability, including visual impairment, motor and sensory deficit with limited ambulation and manual dexterity, pain and bowel/bladder dysfunction [3,12].

Early and appropriate diagnosis of NMO and institution of treatment is important in view of the relapsing-progressive course of the disease, its severe damage to the nervous system and its devastating impact upon patients’ quality of life. Differentiating NMO from MS is particularly relevant, because disease-modifying therapies, effectively used in MS, appear to have weak or even adverse effects upon NMO course [13,14]. Recent years have seen enormous progress in the elucidation of the pathogenesis of NMO, with emerging treatment options targeted at specific elements of autoreactive immune response. Several clinical trials have been completed or are being conducted to provide evidence for optimal therapeutic strategies, aimed at suppression of relapse as well as modifying the further course of the disease.

The aim of this study is to review—on the basis of the relevant literature—currently available therapeutic options in NMOSD and future directions of ongoing research in this field.

## 2. Methods

The authors conducted a literature search focused on the topic of treatment in NMOSD. The key search terms applied in PubMed via MEDLINE were “NMO” or “NMOSD” and “treatment”. The online search covered the publication period from database inception, i.e., 2010, until 31 January 2021. Reviews and research studies, classified according to their relevance, were initially included, with subsequent exclusion of conference abstracts and papers written in languages other than English. In addition, reference lists from eligible publications were searched for their relevance to the topic.

## 3. Pathogenesis of NMODS

The main pathological mechanism involved in the background to NMO is associated with autoreactive anti-AQP4 IgG antibodies. Their relevant role has been confirmed in experimental models (NMO pathology was induced in rats after recombinant IgG transfer) as well as in clinical studies (anti-AQP4 IgG level correlated with relapse activity and the extent of spinal cord lesions shown in MRI) [15,16,17].

The immune target, AQP4, is a tetrameric membrane-forming protein constituting a water channel. The highest expression of this protein is observed in the central nervous system, but it is also present in the epithelial cells of the renal tubules, in the gastric parietal cells, in the respiratory tract, endocrine glands and skeletal muscles [17,18,19]. Within CNS, AQP4 can be found in the parenchyma of the brain and spinal cord, in the meninges and in the optic nerve, with some variability in its distribution [20]. A high concentration of AQP4 occurs in astrocytes, especially at their end-feet which adhere to the endothelium of cerebral vessels, contributing to a blood-brain barrier (BBB) function [8]. The shorter isoform of AQP4 (M23) tends to form clusters, so-called orthogonal arrays of particles (OAPs) [3,17].

It has still not been clearly elucidated how the autoimmune attack at AQP4 is initiated. Possible mechanisms include inappropriate recognition of this protein by T-cells or impairment of early B-cell tolerance checkpoints. Complex interplay of T-cells and B-cells in this process includes presentation of relevant antigens, dynamic balance between Th17 and Treg (with downregulation of the latter), secretion of proinflammatory cytokines and stimulation of B-cells expansion and proliferation [8,11,21,22]. Among the range of cytokines, IL-6, released by CD4+ Th cells and astrocytes, is regarded as playing a key role in this autoreactive inflammatory cascade [21,23].

As a result, the autoreactive pool of specific B-cells (CD19+, CD27+, CD180-) differentiates into plasmablasts, producing anti-AQP4 IgG. They enter the CNS due to a non-specific inflammatory environment or penetration through small parenchymal vessels [8,14]. IgG selectively bind to the extracellular loop of AQP4. Their multivalent binding within OAPs further enhances binding affinity for the C1 component, initiating complement activation and the complement-dependent cytotoxicity pathway [24]. These processes are mediated by the activity of anaphylatoxins and membrane-associated regulatory proteins [14,25]. Further disruption of BBB causes extravasation of serum albumin, fibrinogen and immunoglobulins as well as increased influx of macrophages, eosinophils and neutrophils into the CNS tissues [26]. In addition to this inflammatory injury, secretion of excitotoxic compounds and free radicals ultimately accounts for the death of astrocytes, oligodendrocytes and neurons [11,27]. Damage to those cells which do not directly express AQP4 is probably associated with a “bystander” injury within an inflammatory environment, release of glutamate from primarily injured astrocytes or inhibition of water permeability due to water channel dysfunction [14,27].

Pathological findings typical for seropositive NMOSD reveal severe damage to astrocytes and oligodendrocytes, with preferential loss of AQP4, glial fibrillary associated proteins (GFAP) and myelin associated glycoproteins (MAG) showing a vasculocentric pattern. Active demyelinating lesions, also with perivascular distribution, are accompanied by deposition of activated complement components [24,28]. These features of NMOSD-related CNS damage clearly distinguished the disease from MS as well as MOGAD, despite their shared immune-mediated demyelinative background.

## 4. Current NMODS Treatment

Therapeutic approach to NMOSD comprises treatment of acute relapses and long-term maintenance therapy that prevents further exacerbations and accumulation of disability [29] (Table 2).

The treatment of relapses includes intravenous pulses of methylprednisolone (IVMP), plasma exchange (PLEX), intravenous immunoglobulins (IVIG) and immunoadsorption therapy (IA). For maintenance therapy, non-selective immunosuppressants as well as agents with specific immune targets may be considered. In 2014 recommendations were published by the Neuromyelitis Optica Study Group (NEMOS) [29]. Evidence-based findings from ongoing investigations, especially within the last two 2 years, have contributed to updated recommendations, with an increasing repertoire of therapeutic options [30].

### 4.1. Treatment of Relapse

Intravenous pulse of methylprednisolone (IVMP) with or without oral tapering remains the first line of relapse treatment [31]. Its mechanism of action is based on inhibition of the inflammation cascade through suppressed production of inflammatory cytokines and proliferation of monocytes. IVMP should be administered as early as possible, because any delay may substantially affect the relapse outcome. Nakamura et al. showed that early administration of steroids in optic neuritis is associated with protection of the retinal nerve fiber layer and a better outcome in terms of vision impairment [32]. Clinical benefit was also demonstrated for IVMP in the acute phase of myelitis in the course of NMO. High-dose IVMP pulse therapy is recommended especially in patients with AQP4-IgG-positive NMOSD [33]. Several factors, including previous use of immunosuppressants, high levels of CSF protein, and brainstem syndrome with active lesions in MRI and respiratory failure, predispose to poor response to IVMP [4,15]. In such patients escalation of treatment should be considered.

Therapeutic plasma exchange (plasmapharesis PLEX) is an alternative or additive option in relapse treatment. PLEX is based on the extracorporeal blood separation technique designed to remove pathogenic autoreactive antibodies from the systemic circulation [34]. The efficacy of PLEX has been shown for 44–75% NMO patients, especially when undertaken early and during the first episode of relapse. Other factors associated with positive response to PLEX include a shorter disease duration with fewer previous relapses and lower residual disability and clinical manifestation other than optic neuritis [35,36,37]. With the relative safety of the PLEX procedure (mild to moderate complications are about 36%), its benefit outweighs the risk of deterioration of neurological deficit [38].

Immunoadsorption (IA) is a more selective method of apheresis, which allows the removal of specific antibodies and immune complexes from systemic circulation, without total plasma exchange. Findings from retrospective studies [39,40] suggest that PLEX and IA may be more effective in isolated LETM than in the classical NMO phenotype, without significant differences between these procedures. Hoffmann et al. [41] demonstrated that IA is a safe and effective treatment option for pregnant and breastfeeding women with NMO relapse. Patients with isolated myelitis have been shown to respond better to PLEX/IA than IVMP [30].

Another option for NMO relapse treatment is intravenous immunoglobulins (IVIG). The complex mechanism of IVIG includes neutralization of proinflammatory antibodies, inhibition of the complement, alteration of Fc-γ-receptor on monocytes and B cells, but also downregulation in T-cells activation and cytokine secretion. This mode of action seems beneficial particularly for seropositive NMOSD patients, with severe neurological deficit at the onset of the disease [4,15,29]. Apart from reducing symptoms of relapse, IVIG treatment is supposed to provide a longer subsequent remission, although there is still little evidence for this [42,43]. IVIG are safe and well-tolerated [44,45].

### 4.2. Maintenance Treatment

Maintenance therapy should be instituted shortly after treatment of relapse in order to prevent future exacerbations and accumulating disability.

#### 4.2.1. Immunosuppression

Although not officially approved and mostly used on an empirical basis or tested in short-term, open label trials, a few immunosuppressants have been applied in NMO as a single or combined therapy [46].

Traditionally azathioprine (AZA) was used in NMO as the first line treatment. Azathioprine is a purine analogue, transformated in the liver and erythrocytes to the active metabolite, 6-mercaptopurine, which inhibits DNA synthesis and proliferation of B and T cells [47]. AZA is metabolized by thiopurine methyltransferase (TPMT), the activity of which depends of TPMT genetic polymorphism, so persons with a TPMT*3C heterozygous or homozygous genetic mutation are more predisposed to adverse events of treatment. Reduction of relapse rate and stabilization of neurological deficit have been achieved with the use of AZA alone or in combination with corticosteroids [15,48,49]. However, a delayed mechanism of action and poor tolerance are main limitations of this treatment option.

Beneficial effects of NMO treatment with other immunosuppressive agents, including mitoxantrone, methotrexate, cyclophosfamide and cyclosporine, have been confirmed only in case series or limited groups of patients. Their mode of action involves unspecific depletion or diminished activation of cells and factors engaged in the inflammatory process. The safety profile of these medications has to be thoroughly considered, because of possible cardio-, hepato-, pulmonary and bone marrow toxicity [50,51,52,53].

There is more evidence available for the effective use of mycophenolate mofetil (MMF) in NMOSD. This prodrug of mycophenolic acid suppresses proliferation of B and T-cells through inhibition of inosine monophosphate dehydrogenase. Retrospective studies have shown stabilization or improvement in disability level and—to a lesser extent—reduction in the relapse rate in NMOSD patients, without differences between those with seropositive or seronegative status. A prolonged period of effective action and side effects may affect the patients’ adherence to treatment [54,55].

#### 4.2.2. Cell Depletion

Recognition of the principal function of B cell abnormalities and autoreactive humoral response in the etiology of NMO has encouraged the application of B cell depletion therapies.

Rituximab (RTX) is a chimeric monoclonal antibody against the CD-20 antigen, which is expressed on the majority of B-cells, except for their early stages and differentiated plasmablasts. RTX treatment, applied in lymphomas, leucaemia and autoimmune diseases, results in efficient and prolonged depletion of B-cells without overall suppression of immune homeostasis [15]. In several studies a significant reduction of relapse rate and disability measures has been demonstrated in NMO patients treated with RTX, independent of clinical variables and serostatus [56,57]. Better efficacy of RTX in comparison with MMF and AZA has also been observed [58]. Consequently, off-label application of RTX in NMOSD has significantly increased. With overall good tolerability, the risk of severe opportunistic infections (including progressive multifocal leukoencephalopathy) and cardiovascular failure has to be taken into account in monitoring the safety of treatment.

Inebilizumab is a humanized monoclonal antibody against CD-19, a marker expressed on a wide range of B-cell lines. Its action results in sustained depletion of B-cells, through antibody-dependent cell-mediated cytotoxicity [59]. In a phase-3 double blind, randomized trial (N-MOmentum), inebilizumab was found to prolong the time to subsequent NMO relapse and reduce worsening of disability and radiological (MRI) measures of disease activity. The results were even more significant, considering that no immunosuppressive agents were used in this trial as comparators or combined therapy. Treatment was well tolerated, with the incidence of adverse events comparable to the placebo [60]. Thus, in 2020 inebilizumab was approved by the FDA for treatment in NMOSD seropositive patients [61].

#### 4.2.3. IL-6—Targeted Activity

Interleukin-6 (Il-6) plays an important role in regulation of the balance between T17 and Treg activity and initiating inflammatory cascade which ultimately induces anti-AQP4 IgG production by a subpopulation of B cells. Levels of Il-6 and Il-6 receptors are significantly elevated in CSF and serum during NMO relapses and they correlate with clinical and radiological measures of disease activity. Thus, Il-6 and Il-6 receptors seemed a promising target for novel therapies [21,62].

The first Il-6 receptor antagonist adapted for NMO treatment was tocilizumab, a humanized monoclonal antibody [63], previously used in therapy of autoimmune arthritis. Some beneficial effects were shown after administration of tocilizumab in small groups of NMO patients, including those not responding to cell depletion therapies [64]. An open-label, multicenter, randomized phase 2 trial (TANGO) [65] demonstrated the advantage of tocilizumab over AZA in achieving clinical endpoints (reduced relapse rate and sustained disability), with a small percentage of severe outcomes and a mild increase in transaminases as the main adverse effects [29,64,66]. The subcutaneous form of tocilizumab appeared to have similar efficacy to intravenous infusion, with apparently better convenience for patients [67].

Satralizumab is a humanized recombinant monoclonal antibody targeting the Il-6 receptor. A particular design of this molecule allows “recycling antibody technology” (after degradation of Il-6 R in the endosome acidic environment, the antibody dissociates from this complex and is released into plasma, resuming high binding affinity to Il-6 R) which influences the pharmacokinetics of the drug and enhances its efficacy. Two phase 3, double-blind, randomized multicenter trials have demonstrated beneficial effects of satralizumab upon NMO clinical course, when used as additive treatment to baseline low-dose immunosuppressants (SAkuraSky) or as a monotherapy (SAkuraStar). In both trials clear differences in the disease outcomes were observed between subjects who were seropositive and seronegative for anti-AQP4 Ig, with a disadvantage for the latter. The safety profile and tolerability of satralizumab were good, without remarkable adverse events [16,68]. In 2020 satralizumab was also approved by the FDA as the therapy for seropositive NMO patients.

#### 4.2.4. Complement Inhibition

Complement cascade and membrane attack complex, engaged in inflammatory injury of astrocytes and neurons, created another putative target for NMO therapies.

Eculizumab is a humanized monoclonal antibody, targeted at terminal complement protein C5 and preventing its cleavage into C5a and C5b components. Initially used in rheumatological inflammatory diseases like lupus erythematosus, rheumatoid arthritis and paroxymal nocturia hemoglobinuria, paroxysmal nocturnal hemoglobinuria and myasthenia, eculizumab has been shown to suppress NMO development in experimental models [69,70]. In the PREVENT randomized double-blind trial, AQP4-IgG seropositive patients treated with eculizumab (mostly with continuation of prior immunosuppression) had a significantly lower risk of relapse than those who received a placebo. However, no differences were found in measures of disability progression. Due to its mode of action, eculizumab increases the risk of bacterial infections as adverse effects, so appropriate prevention has to be considered [71]. Initial clinical data on the efficacy of eculizumab in treating NMOSD come from a small open-label study from Pittock and colleagues in 2009/2010. The first controlled clinical trial published in 2013 proposed that eculizumab may prevent the relapse of NMOSD [70].

Also, a phase II/III patient pediatric study, a study safety and activity of eculizumab in pediatric participants with relapsing NMOSD” has been underwent since January 2020 [72]. Eculizumab is well tolerated, significantly reduce frequency of relapse attack, stabilise neurological state in patients with NMOD, especially in patients with aggresive forms of the disease [9,73,74]. In 2019, the FDA and EMA approved eculizumab for the treatment of seropositive NMOSD patients with a relapsing course of disease [19].

## 5. Future Therapies

Directions of research in the field of future therapies for NMO include further exploration of already known mechanisms, as well as investigation of potential novel targets.

### 5.1. Cell Depletion

Ublituximab is a new monoclonal antibody against CD20 antigens, which has demonstrated B cell depletion and clinical improvement in a phase I open clinical trial with seropositive NMOSD patients [75,76]. Another monoclonal antibody under investigation, belimumab, is targeted against the B-cell activation factor (BAFF). B-cell depletion might also be achieved through adoptive transfer of tandem chimeric antigen receptor (CAR) T-cells—therapeutic intervention developed for hematological neoplasms, which has been tested on animal models of NMO [77].

### 5.2. Complement Inhibition

There are ongoing pre-clinical or clinical trials investigating the efficacy of agents which target different components of the complement pathway (e.g., C1, C3, C5, properdin). An alternative approach is associated with upregulation of the complement regulatory complex (membrane-associated glycoproteins); in some studies statins have been shown to express such an action [78].

### 5.3. Counteracting Anti AQP4 IgG

Aquaporumab is a humanized recombinant monoclonal antibody which competes with pathogenic IgG in binding AQP4. In comparison with pathogenic antibodies, aquaporumab demonstrates greater affinity to its target, but due to Fcγ mutation does not activate complement and cell-dependent toxicity. Its efficacy has been shown in preclinical studies [79].

Deglycosylation or cleavage of antiAQP4 IgG deprives the antibodies of their pathogenic activity and prevents binding to the target. Bacteria-derived enzymes with such properties may be applied by therapeutic apheresis or intravenous infusion [14].

Other putative modes of action for novel therapies include inhibition of neonatal Fc receptor, which protects pathogenic IgG from their degradation in lysosomes, or their elimination from plasma with the use of highly selective immunoadsorption [14].

### 5.4. Polynuclear-Targeted Activity

Recently, the pro-inflammatory role of neutrophils and eosinophils infiltrating CNS tissues within NMO-related lesions has been highlighted, making these cells another promising target of therapeutic interventions.

Inhibition of eosinophils may be achieved with the use of monoclonal antibodies against Il-5 such as mepolizubam or reslizumab, or histamine H1 receptor antagonist—cetirizine, which has been found to be effective in a pilot clinical trial [80,81]. Sivelestat, tested on animal models of NMO, is a neutrophil protease inhibitor, aimed at reduction of inflammatory cytokine production and neutrophil-induced capillary permeability [82,83].

### 5.5. Other Avenues

The inhibition of the proteasome decreases the degradation of regulatory molecules engaged in the cell cycle. Bortezomib is a selective proteasome inhibitor, which enhances apoptosis of plasma cells secreting AQP4-Ab and reduces lymphocyte proliferation. Previously used in hematological neoplasms, bortezomib has shown efficacy in small clinical trials with NMO, including patients refractory to other therapies [84,85].

Active endothelial proteins (glucose-related protein—GRP78) or monoclonal antibodies against the vascular endothelial growth factor (bevacizumab) are considered to be factors potentially preventing BBB disruption, a relevant element in NMO background [26].

Another potential target for NMO treatment is associated with restoration of immune tolerance and suppression of autoreactive T-cells. Investigated options include inverse DNA or autoreactive T-cell vaccinations and therapies based on Treg cells and tolerogenic dendritic cells [14]. Hematopoietic stem cell transplantation (HSCT) is also being explored in this field, with improvement in clinical outcomes and immunological measures of NMO observed mainly after allogeneic HSCT [86,87,88,89].

Attempts to repair demyelinative NMO-related damage to CNS have already been explored with regard to multiple sclerosis. Remyelination is supposed to be stimulated by differentiation and proliferation of oligodendrocyte precursor cells, and such an effect is observed with the use of opicinumab (LINGO 1 blocker) and clobetasol [90].

## 6. Conclusions

In recent years, research studies have provided better insights into the background of NMOSD and have led to great progress in our understanding of the nature of the disease, followed by extensive introduction of new treatment options. In long-term preventive therapy, immunosuppressants have been gradually replaced by agents selectively targeted at specific elements of the autoimmune cascade. These include drugs adapted from treatment regimens of neoplasms or other autoimmune disorders, as well as newly designed molecules. Instead of empirical or “off-label” use, there is an increasing trend for recommending officially approved therapies based on evidence from randomized controlled trials.

Despite this undoubted progress in the field of NMOSD treatment, there are still some challenges ahead. Insufficient response to treatment in seronegative patients and limitations associated with adverse effects of some drugs show the need for further investigation, exploring novel mechanisms and potential targets for therapeutic intervention.

New perspectives in NMOSD raise the hope for satisfactory management of this severe and disabling disease, as well as for therapeutic implications for other neurological disorders with an autoimmune background.

## Figures and Tables

**Table 1:** Criteria for the diagnosis of NMOSD [6].

**Core Clinical Symptoms**	Optic neuritis Acute myelitis Area postrema syndrome—unexplained hiccups, nausea or vomiting Acute brainstem syndrome (oculomotor disturbances, bulbar syndrome, respiratory failure) Symptomatic narcolepsy or acute diencephalic syndrome (apathy or agitation, hypersomnia, obesity, autonomic dysfunction) with NMOSD-typical changes in MRI Symptomatic cerebral syndrome (confusion, seizures) with NMOSD-typical brain lesions
**NMOSD with AQP4-IgG Positive**	At least 1 core clinical symptom Positive AQP4-Ab-IgG test Exclusion of any other diagnosis
**NMOSD with AQP4-IgG Negative or Unmarked**	At least 2 core clinical symptoms present as a result 1 or more clinical attacks of the following: - at least 1 core clinical symptom must be optic neuritis, acute myelitis with LETM or area postrema syndrome - dissemination in space (2 or more core clinical symptoms) - fulfillment of additional MRI criteria AQP4-IgG negative or test unavailable Exclusion of any other diagnosis
**MRI Criteria for NMOSD without AQP4**	Acute optic neuritis: - no change or non-specific changes in the white matter of the brain OR - optic nerve with T2-hyperintense lesion or T1-weighted gadolinium enhancing lesion extending over >1/2 optic nerve length or involving optic chiasm Acute myelitis: - intramedullary MRI lesion extending over ≥3 contiguous segments (LETM) OR - ≥3 contiguous segments of focal spinal cord atrophy in patients with history compatible with acute myelitis Area postrema syndrome Acute brainstem syndrome

**Table 2:** Currently available therapies for NMOSD. (* drugs approved by FDA/EMA) [29].

Name	Route	Dosing Regimen	Mode of Action
Treatment of Acute Relapse			
methylprednisolone	iv	1000 mg for 3–5 days	multiple anti-inflammatory
plasma exchange	iv	5–7 cycles	removal of auto-antibodies and inflammatory cytokines
immunoadsorption	iv		removal of auto-antibodies and inflammatory cytokines
intravenous immunoglobulin	iv	0.4 g/kg/day with 5 days	multiple anti-inflammatory
**Preventive Treatment**			
azathiopryne	oral	2 to 3 mg/kg/day	Immunosuppressant, depletion of B-cells and T-cells
mitoxantrone	iv	12 mg/m^2^ every 3 month (max dose 140 mg/m^2^)	anthracenedione antineoplastic agent, intercalates DNA
metotrexate	oral	7.5–25 mg weekly	folic acid inhibitor, modulation of T cells activity
cyclofosfamide	iv	2 g daily for 4 days	alkylating agent, inhibits white blood cells
cyclosporine A	oral	2–5 mg daily	calcineurin inhibitor, inhibits T-cells
mycophenolate mofetil	oral	750–3000 mg daily	immunosupresant inhibitor of inosine monophosphate dehydrogenase, depletion and suppressed proliferation of B and T cells
rituximab	iv	1 g on days 1 and 14; repeated every 6 months	chimeric monoclonal antibody anti CD20
inebilizumab *	iv	300 mg on days 1 and 15	humanized monoclonal antibody anti-CD19
tocilizumab	iv	8 mg/kg every 4–6 weeks	recombinant humanized monoclonal antibody anti lL-6 receptor
satralizumab *	sc	120 mg	humanized monoclonal antibody anti Il-6 receptor
eculizumab *	iv	900 mg weekly for 4 weeks	recombinant humanized monoclonal antibody anti-C5

## Data Availability

The data presented in this study are available upon request from the corresponding author. The data are not publicly available.

## Abbreviations

AQP4	aquaporin 4
AZA	azathioprine
BBB	blood-brain barrier
CNS	central nervous system
CSF	cerebrospinal fluid
ECP	eosinophil cationic protein
GFAP	glial fibrillary associated protein
HSCT	hematopoietic stem cell transplantation
IA	immunoadsorption
IgG	immunoglobulin G
IVIG	intravenous immunoglobulins
IVMP	intravenous pulse of methylprednisolone
LETM	longitudinal extensive transverse myelitis
MAG	myelin associated glycoprotein
MBPE	eosinophilic granule major basic protein
MMF	mycophenolate mofetil
MRI	magnetic resonance imaging
MOG	myelin oligodendrocyte glycoprotein
MS	multiple sclerosis
NMOSD	neuromyelitis optica spectrum disorders
OAPs	orthogonal arrays of particles
ON	optic neuritis
PLEX	plasmapharesis
RTX	rituximab
TPMT	thiopurine methyltransferase
